# Longitudinal Extensive Transverse Myelitis due to Varicella-Zoster Virus Infection in an Undiagnosed HIV-Positive Patient

**DOI:** 10.1155/2024/9027198

**Published:** 2024-07-18

**Authors:** Elahe Yaghmaei, Ahmad Najafi, Reza Daneshvar Kakhki

**Affiliations:** ^1^ Department of Neurology Kashan University of Medical Sciences, Kashan, Iran; ^2^ Infectious Diseases Research Center Kashan University of Medical Sciences, Kashan, Iran; ^3^ Autoimmune Diseases Research Center Kashan University of Medical Sciences, Kashan, Iran

## Abstract

**Introduction:**

Longitudinal extensive transverse myelitis (LETM) has four main causes: inflammatory, malnutrition, vascular, and infectious causes. Among the commonly described viral causes leading to LETM are the *Herpesviridae* family, HIV, and HTLV-1. *Case Presentation*. A 43-year-old man presented with asymmetric weakness of the lower limbs (the left side was weaker), urinary retention, and flank pain. The symptoms began five days after shingle eruption and progressed over twelve days. He was diagnosed with longitudinal extensive transvers myelitis extending from T4 to T6, which corresponded to the same dermatome involved in shingles. The PCR result of cerebrospinal fluid was positive for varicella-zoster virus with a viral load of 500 copies/ml. Additionally, the initial HIV enzyme-linked immunosorbent assay (ELISA) test was positive, and his CD4 count was 72 cells/mm^3^. Other lab results were normal. Based on the appearance of LETM in the thoracic MRI at T4-T6, VZV myelitis was diagnosed, and treatment was initiated with acyclovir (30 mg/kg divided daily for twenty-one days), methylprednisolone (1 g/day for three days), prophylactic antibiotics (trimethoprim/sulfamethoxazole, rifampin, and isoniazid), and antiretroviral therapy (dolutegravir and Truvada). After 2-month follow-up, he was nearly free of symptoms.

**Conclusion:**

Infection is one of the critical causes of transverse myelitis. When a patient presents with skin shingles along with myelopathy, varicella-zoster myelitis should be considered, and the patient should be evaluated in terms of immune system dysfunction. Treatment with acyclovir has been shown to be effective in reducing clinical symptoms in such cases.

## 1. Introduction

Longitudinal extensive transverse myelitis (LETM) has four leading causes: inflammatory (including demyelinating disorders, systemic collagen vascular diseases, and paraneoplastic syndromes), malnutrition (such as vitamin B12 and copper deficiencies), vascular (including dural arteriovenous (AV) fistulas and infarction), and infectious causes [1, 2].

The commonly described viral causes leading to LETM include the *Herpesviridae* family (varicella-zoster virus, herpes simplex, Epstein–Barr virus, and cytomegalovirus), HIV, and HTLV-1 [2].

Reactivation of varicella-zoster virus in the posterior root ganglion of the spinal cord, trigeminal ganglion, and other cranial nerves can causes shingles. It is diagnosed by a painful rash and blisters in one or more adjacent dermatomes. In patients with immune system deficiencies, skin involvement may be observed in multiple dermatomes, with bilateral involvement, or with spread to the central nervous system [3, 4]. Central nervous system involvement can lead to meningitis, ventriculitis, encephalitis, and, less frequently, stroke or myelitis [4].

A rare complication of varicella-zoster reactivation is shingles myelitis [4]. It may occur at the same time or up to a few weeks after the appearance of a skin rash, or even in the absence of a skin rash. Various mechanisms, including inflammatory, vasculitis, or even direct invasion of the virus into the spinal cord, may play a role in its occurrence [5].

## 2. Case Presentation

A 43-year-old man presented with asymmetric weakness of the lower limbs (the left side was weaker), urinary retention, and flank pain. Muscle weakness and pain began 12 days before hospitalization (5 days after shingles erupted on the right side), and urinary retention started the day before hospitalization. On the admission day, his blood pressure was 110/75 mmHg, heart rate was 75 Beat/min, and body temperature was 36.8°C. He had crusted lesions in the T6-7 dermatome region on the right side. The rest of the systemic examination was normal. He was alert and oriented, and cranial nerve examination and fundoscopy were normal. The force of the upper limbs was normal. Both proximal lower limbs were asymmetrically weak (left side more than right side), rendering him unable to stand or walk without aid. He also exhibited mild weakness in the distal portions of both lower limbs. Pain perception decreased up to dermatome T7 on the right side and around T5 on the left side, with similar levels of decreased sweating and pain perception on both sides. The patient's plantar reflex on both sides was extensor and deep tendon reflexes of the lower limbs were slightly deceased slightly and almost symmetrical. He did not mention any history of previous illness or visit to a doctor. He hailed from Afghanistan, was single, and had been living in Iran for a year. The patient worked as a construction worker.

**Figure 1:**
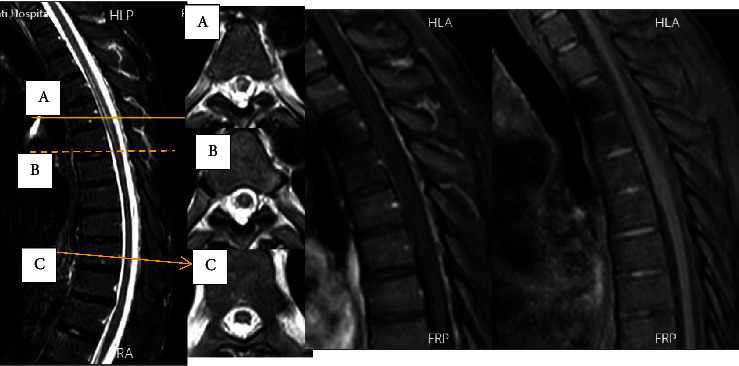
Thoracic Cord Imaging. The leftmost image represents the midsagittal section of the thoracic cord. The subsequent series of pictures display axial views of three distinct segments of the spinal cord (labeled as A, B, and C) corresponding to the midsagittal section. The third image depicts the T1-weighted sagittal section without contrast injection. The image on the right shows the T1-weighted sagittal section after gadolinium injection.

**Figure 2:**
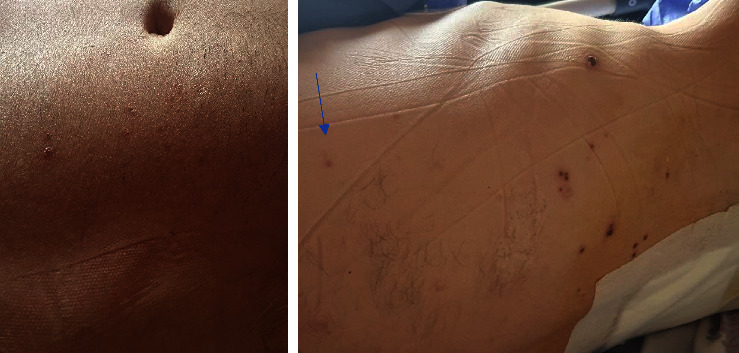
Image of the patient's rash around dermatomes T8-9 showed crusted lesions and some pustular lesions, as seen around dermatome T5 (indicated by the arrow).

**Figure 3:**
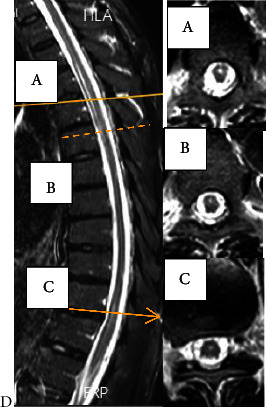
Thoracic MRI after 12 days of treatment. Lesion is better visible around T4-T6 (A and B) and T11-T12 (C).

He exhibited a triad of myelopathy, prompting a request for a total spinal cord MRI. Due to the appearance of LETM on the thoracic MRI at T4-T6 (Figure 1), which corresponded to the same dermatome involved in shingles (Figure 2), and the positive result of cerebrospinal fluid PCR for varicella (with 500 copies/ml), the diagnosis was shingles myelitis. Treatment with acyclovir (600 mg intravenous every 8 hours) and dexamethasone (4 mg intravenous twice daily) was initiated, and evaluation for immunosuppression began. The initial serum HIV ELIZA test was positive, and the viral load checked was positive (1800 copy/ml). His CD4 count was 72 cell/mm^3^. The results of the tuberculosis assessment, which included chest radiography and purified protein derivative (PPD) testing, were found to be negative. Other lab results are detailed in Table 1. Treatment with dexamethasone was discontinued. To control AIDS, he started taking dolutegravir and Truvada (emtricitabine and tenofovir) daily. Due to a positive *Brucella* IgG test, he received treatment with trimethoprim/sulfamethoxazole 800/160 BD and rifampin 600 daily for 42 days, along with isoniazid 150 mg per day and vitamin B6 to prevent tuberculosis. On the seventh day after starting acyclovir, the patient's muscle strength significantly improved to 4+/5, and he could walk without aid. However, even after removing the internal Foley catheter, he still experienced urinary and fecal incontinence. Blood cultures, urinalysis and culture, and the chest X-ray yielded negative results for infection. Due to persistent urinary retention, the patient received treatment with methylprednisolone 500 mg twice a day for three days. Unfortunately, no immediate sphincter improvement occurred. A reevaluation by MRI requested twelve days after the first MRI imaging revealed LETM in the previous location with some evidence of myelomalacia and no signs of progression (Figure 3). After two months of follow-up, he regained near-normal gait and urinary continence.

## 3. Discussion

Our patient's symptoms started with shingles in the thoracic region. Within five days after the onset of skin symptoms, he had paraparesis, which progressively worsened over ten days, followed by sphincteric symptoms. When a patient with myelopathy presents with fever, confusion, meningismus, rash, concomitant systemic infection, and lymphadenopathy or resides in an endemic area for parasitic infections, suspicion of infectious myelopathy increases [6]. Due to the similar clinical presentation and CSF pattern, distinguishing neuromyelitis optica spectrum disorders from direct infectious myelitis is often challenging. The most reliable evidence of direct infection occurs when a pathogen is isolated from cerebrospinal fluid (CSF), when PCR results are positive, or when both acute and convalescent CSF and serum antibody titers demonstrate a significant increase or change. This suggests an ongoing or recent infection [6].

It is uncommon for immunocompetent patients to develop myelopathy as a result of varicella-zoster infection, although there are a few documented cases [3]. So, it is necessary to carefully evaluate the immune system deficiency in these patients. A positive HIV test established immunodeficiency of our patient.

The most significant risk of neurologic complications of disseminated herpes zoster is in patients with HIV whose CD4 cell count is less than 200 cells/mm^3^ [7]. Varicella-zoster virus causes different neurological syndromes in patients with immunodeficiency, including multifocal leukoencephalopathy, CNS vasculitis, ventriculitis, myelitis and myeloradiculitis, optic neuritis, cranial nerve palsies, focal brain-stem lesions, and aseptic meningitis [4].

In our case, based on the typical manifestations of myelopathy, MRI findings of the spinal cord, history of shingles skin scarring before the onset of symptoms, and the detection of virus DNA by PCR in the cerebrospinal fluid, LETM was diagnosed as a result of varicella-zoster virus infection.

There is no agreed-upon standard for treating myelitis caused by varicella zoster in patients with weakened immune systems. A PubMed search from 1992 to December 2023 revealed only a few cases of VZV myelitis in HIV-positive patients, with information on their treatment plan and long-term outcome.

De La Blanchardiere et al. conducted a multicenter case series of 34 HIV-positive patients with varicella zoster-related neurological complications. Among them, only eight had myelitis, which was associated with a high rate of mortality and severe neurological sequelae (72%), compared to other neurological manifestations (27%). The treatment methods varied across the patients, and the authors did not specify the treatment for myelitis cases [8].

Lameiras et al. reported a case of shingles rash, fever, paraparesis, sphincteric disorder, and meningismus. MRI detected a LETM lesion, and PCR confirmed VZV in the cerebrospinal fluid. The patient was treated with acyclovir (10 mg/kg) and dexamethasone 4 mg three times daily for a month, followed by an oral prednisolone taper starting at 20 mg for another month. After the treatment, the patient experienced brief motor recovery [9]. Our patient had a lower CD4 level, a lower varicella viral load, mild CSF pleocytosis, no meningismus signs, less severe initial symptoms, and a faster and more complete recovery than Lameiras' case. This suggests that the severity of the immune system response has a greater influence on the spinal cord inflammation and damage than the viral load. The only major difference in treatment was the administration of a high-dose corticosteroid injection (methylprednisolone) in our patient versus low-dose corticosteroid injection in their patient, which might have affected the final outcome.

Weiss et al. described a case of VZV myelitis in an HIV-positive patient with poor adherence to antiretroviral therapy. The CSF HIV viral load was 1,030 copies/mL, suggesting “secondary” HIV CSF escape. She underwent intravenous acyclovir treatment for four weeks and was followed by oral famciclovir, then valacyclovir for six weeks. She also got dexamethasone to reduce inflammation. They reported a significant improvement in motor symptoms in their patient, but she still exhibited some weakness in the affected limb after six months of follow-up [10], whereas our patient had no residual motor symptoms after two months.

Falcone et al. described a case of AIDS manifesting as VZV meningomyeloradiculitis. He had positive CSF-VZV PCR. He received methylprednisolone and acyclovir for 14 days and then switched to famciclovir, which aggravated his symptoms. He resumed acyclovir for four weeks and then continued with valacyclovir for eight weeks, which eventually improved his motor symptoms significantly [11]. This case highlights the importance of long-term antiviral treatment and suggests that the favorable motor prognosis of the patient is related to the effective anti-inflammatory treatment in the beginning.

Stefan Polen described a patient who developed paraplegia in the lower limbs and weakness in the upper limbs within three days, three weeks after having zoster rash and histoplasmosis. The patient was given ganciclovir for five days (then switched to acyclovir for 36 days) and briefly improved in motor symptoms but had a seizure 12 days after starting treatment, which MRI revealed as encephalitis and worsening of cord involvement. The patient received methylprednisolone pulse (one gram daily) for five days and then intravenous dexamethasone (8 mg/daily) for eleven days, followed by oral prednisolone (80 milligrams daily, slowly tapered) for five weeks. During this time, the patient's motor symptoms deteriorated, and MRI showed that brain inflammation had subsided, but extensive intramedullary hematoma had formed at the previous myelitis site in the neck [12]. This case suggests that the delayed anti-inflammatory treatment could not prevent the inflammatory vasculopathy induced by the virus, and that the effective delay dose of anti-inflammatory treatment did not improve the patient's final outcome.

A patient with HIV, who contracted Crohn's disease after shingles, also experienced myelitis zoster. He received acyclovir for 14 days and pulse methylprednisolone (1 gram daily) for five days. Although the patient had a partial recovery at discharge, the long-term prognosis was not reported. These cases suggest that short-term treatment with corticosteroid and acyclovir may be ineffective in improving the short-term prognosis [13].

Bagat et al. described an HIV-positive patient with AIDS who developed paraparesis and urinary and fecal sphincter impairment after zoster involvement in the sacral region. The patient's myelitis slowly improved with acyclovir and dexamethasone (44 milligrams daily), but during hospitalization, he experienced unexplained fevers and eosinophilia. Extensive clinical tests eventually led to the diagnosis of Drug Reaction with Eosinophilia and Systemic Symptoms (DRESS). The patient improved upon discontinuation of acyclovir [14].

In recent years, a new treatment method has been proposed. Some viral diseases may improve with superinfection, which blocks or weakens the primary virus. For example, chickens and mice coinfected with IBDV and another virus had better outcomes than those infected with only one virus [15]. This concept may also apply to VZV infection in HIV patients, but more research is necessary.

The primary and most effective method for preventing infection is through vaccination. While the use of weakened virus vaccines is not recommended for patients with immune system deficiencies, there has been recent success with recombinant vaccines in this patient population. These recombinant vaccines have proven highly effective in preventing shingles and mitigating other complications associated with varicella zoster infection, including vasculitis, disseminated disease, ophthalmic issues, neurological complications, and visceral manifestations [16].

For people with HIV who have severe or complicated varicella, it seems the initial treatment recommendation is intravenous acyclovir 10 mg/kg every 8 hours for 4 weeks.

Currently, there is insufficient conclusive pathological evidence directly linking acute myelitis to varicella zoster infection. In a case report during the subacute stage of the disease, a patient with shingles neuralgia exhibited a hyperintense lesion in the MRI region corresponding to the affected dermatome in the spinal cord. Pathological examination revealed the presence of CD68+ macrophages and CD8+ lymphocytes but no signs of demyelination, axonal loss, or vasculitis [17]. In another case series involving nine postmortem patients, five exhibited either focal or diffuse necrosis, while two displayed mild inflammation. Additionally, two patients displayed cord thinning and mild vascular proliferation [18]. Based on the pathology findings, early corticosteroid treatment before the onset of necrosis and vasculitis changes appears more effective. However, initiating anti-inflammatory treatment after the development of vasculitis and hemorrhagic necrosis may not significantly impact the long-term prognosis.

Although in immunocompromised patients, the initiation of corticosteroids is associated with a higher risk of opportunistic infections due to the increased risk [19], early treatment with a high dose of methylprednisolone appears to be more effective in reducing neurologic sequelae.

Delayed diagnosis, a high varicella zoster viral load in CSF, and severe initial inflammatory symptoms appear to be risk factors for poor neurologic recovery.

## 4. Conclusion

One of the critical causes of transverse myelitis is infection. In a patient who has skin shingles along with myelopathy, varicella-zoster myelitis should be considered, and the patient should be evaluated for immune system dysfunction. Treatment with acyclovir and corticosteroids is effective in reducing the patient's clinical symptoms.

## Figures and Tables

**Table 1:** Laboratory data.

*Serum*	
WBC	5.8 thousand cells/mcL
RBC	4.65 million cells/mcL
HB	14.1 mg/dl
MCV	85.38 femtolitre
PLT	169 thousand cells/mcL
Neutrophils	82.5%
Lymphocytes	10.1%
Creatinine	0.9 mg/dl
ESR (1 hour)	20 mm/hour
CRP	10 mg/L
*Brucella* IgM titer	1.9
*Brucella* IgG titer	12.6
Wright, combs wright and 2 ME	Neg
HBs Ag	Neg
HBc AB	11.8 mIU/mL
HCV	Neg
VDRL	Neg
COVID PCR (nasopharynx)	Neg

*Urine analysis*	
Color	Yellow
PH	8
Protein, glucose, ketone, nitrite	Neg
Blood	3+
RBC	55–60 cells/mcL
WBC	1-2 cells/mcL
Ep	2-3 cells/mcL
Culture (72 h)	Negative
Culture (72 h)	Negative

*CSF analysis*	
Pressure	180 mm water
Protein	104 ^mg/dl^ (serum total Pr.^ỻ^ = 7.1 ^gr/l^)
Glucose	87 ^mg/dl^ (^*∗*^BS 172 ^mg/dl^)
RBC	50 cells/mcL
WBC	120 cells/mcL
Neutrophil	20%
Mononuclear	80%
HSV PCR	Negative
CMV PCR	Negative

^ỻ^Serum total protein measured at the same time as CSF puncture. ^*∗*^BS = blood sugar measured at the same time as CSF puncture. mIU/mL = milli-international units per milliliter. mcL = microliter.

## Data Availability

Additional raw data, including radiology and laboratory results, can be provided upon request and with the approval of the research vice president of Kashan University of Medical Sciences.

## Abbreviations

LETM: Longitudinal extensive transverse myelitis
HIV: Human immunodeficiency virus
HTLV-1: Human T-lymphotropic virus type 1
PCR: Polymerase chain reaction
CMV: Cytomegalovirus
EBV: Epstein–Barr virus
VZV: Varicella-zoster virus
CD4: Cluster of differentiation 4.

## References

[1] Kitley J. L., Leite M. I., George J. S., Palace J. A. (2012). The differential diagnosis of longitudinally extensive transverse myelitis. *Multiple Sclerosis*.

[2] Douglas A. G., Xu D. J., Shah M. P. (2022). Approach to myelopathy and myelitis. *Neurologic Clinics*.

[3] Lee J. E., Lee S., Kim K. H. (2016). A case of transverse myelitis caused by varicella zoster virus in an immunocompetent older patient. *Infect Chemother*.

[4] Gershon A. A., Breuer J., Cohen J. I. (2015). Varicella zoster virus infection. *Nature Reviews Disease Primers*.

[5] Arenaza-Basterrechea N., González Fernández J., Al Kassam Martínez D. (2018). Parainfectious longitudinally extensive transverse myelitis associated with varicella-zoster virus. *Neurologia*.

[6] Jacob A., Weinshenker B. G. (2008). An approach to the diagnosis of acute transverse myelitis. *Seminars in Neurology*.

[7] Ku H.-C., Tsai Y.-T., Konara-Mudiyanselage S.-P., Wu Y.-L., Yu T., Ko N.-Y. (2021). Incidence of herpes zoster in HIV-infected patients undergoing antiretroviral therapy: a systematic review and meta-analysis. *Journal of Clinical Medicine*.

[8] De La Blanchardiere A., Rozenberg F., Caumes E. (2000). Neurological complications of varicella-zoster virus infection in adults with human immunodeficiency virus infection. *Scandinavian Journal of Infectious Diseases*.

[9] Lameiras C., Patrocínio de Jesus R., Flor-de-Lima B., Silva J., Pacheco P. (2022). A case of varicella-zoster virus meningomyelitis in an HIV-1-Infected patient: facing the challenges related to its management and prognosis. *Cureus*.

[10] Weiss J. J., Spudich S., Barakat L. (2021). VZV myelitis with secondary HIV CSF escape. *BMJ Case Reports*.

[11] Falcone E. L., Adegbulugbe A. A., Sheikh V. (2013). Cerebrospinal fluid HIV-1 compartmentalization in a patient with AIDS and acute varicella-zoster virus meningomyeloradiculitis. *Clinical Infectious Diseases*.

[12] Pohlen M. S., Sunwei Lin J., Wang K. Y., Ghasemi-Rad M., Lincoln C. M. (2017). Haemorrhagic conversion of infectious myelitis in an immunocompromised patient. *BMJ Case Reports*.

[13] Santos D. H., Carneiro de Oliveira R. d M., Junior W. R. F., Olivetti B. C. (2021). Myelorradiculitis due to Varicella Zoster (Elsberg syndrome) as the first symptom of HIV in a patient with Crohn’s disease in use of Infliximab. *Multiple Sclerosis and Related Disorders*.

[14] Bhagat Y. V., Yunasan E., Alzedaneen Y., Muttana S., Michael M. B. (2021). Treatment of elsberg syndrome causes fever of unknown origin attributable to Drug reaction. *Cureus*.

[15] Bakacs T., Chumakov K., Safadi R., Kovesdi I. (2022). Editorial: fighting fire with fire: using non-pathogenic viruses to control unrelated infections. *Frontiers in Immunology*.

[16] Molero García J. M., Moreno Guillén S., Rodríguez-Artalejo F. J. (2023). Status of herpes zoster and herpes zoster vaccines in 2023: a position paper. *Revista Española de Quimioterapia*.

[17] Moshayedi P., Thomas D., Rinaldo C. R. (2018). Subacute histopathological features in a case of varicella zoster virus myelitis and post-herpetic neuralgia. *Spinal Cord Ser Cases*.

[18] Devinsky O., Cho E. S., Petito C. K., Price R. W. (1991). Herpes zoster myelitis. *Brain*.

[19] Damba J. J., Laskine M., Peet M. M., Jin Y., Sinyavskaya L., Durand M. (2022). Corticosteroids use and incidence of severe infections in people living with HIV compared to a matched population. *Journal of the International Association of Physicians in AIDS Care*.

